# My Neighborhood Is Fuzzy, Not Hard and Fast: A Mixed-Methods Study of Neighborhood Size Among Aging Americans

**DOI:** 10.1093/geroni/igaa057.2471

**Published:** 2020-12-16

**Authors:** Jessica Finlay, Joy Bohyun Jang, Michael Esposito, Sandra Tang, Anam Khan, Philippa Clarke

**Affiliations:** University of Michigan, Ann Arbor, Michigan, United States

## Abstract

In this exploratory sequential mixed-methods study, interviews with 125 adults aged 55-92 living in the Minneapolis (Minnesota) metropolitan area suggest that neighborhood boundaries are “fuzzy”. Qualitative analysis of neighborhood perceptions identified race, mobility, driving status, social connections, housing insecurity, land use, urbanicity, and crime as key themes. Over 8,000 REGARDS participants (mean age 72) indicated how many blocks composed their neighborhoods (mean=9.9, SD=35.4). Linear regression models showed that being over the age of 85, white, less educated, lower income, less physically and cognitively healthy, and living outside of a metropolitan area significantly predicted smaller self-reported neighborhood sizes. Further, there was significant variation among participants residing in the same areas as other respondents. The mixed-methods results indicate that neighborhoods are fluid and dependent on a mix of personal and geographic factors. Findings inform the scale of environmental audits, place-based interventions, and community outreach programs targeting older adults.

